# Résumé of the Cholera of 1867

**Published:** 1868-01

**Authors:** 


### Selections.

### RÉSUMÉ OF THE CHOLERA OF 1867.

Dr. John C. Peters read a very able and interesting paper upon the progress and distribution of the late epidemic, tracing it from its indigenous source, to those portions of the globe where it may more properly be considered as exotic. The various routes pursued by cholera, and their immediate connection with the great lines of commerce and travel, were amply and clearly illustrated by very carefully prepared maps. As an abstract of this paper would scarcely do justice either to its author, or the subject matter, we present the greater portion of it in full, hardly knowing where to compress, since the whole paper itself is a condensed statement of interesting facts, logically and clearly connected.

The Dr. remarked that cholera has prevailed in few places this year; we have merely had to deal with the remains and dregs, as it were, of the great epidemic of 1865, rather than with any new infection, except perhaps in one place, viz., Hurdwar, in Hindoostan, near the source of the Ganges. The great epidemic of 1865, was particularly noted for the course or line of travel which it pursued, for this differed from all previous routes. It started from Bombay, passed up the Red Sea to Mecca, from thence to the Mediterranean, and from thence to all parts of Southern Europe.

Before the establishment of the overland route to India, and the use of steam-vessels on the Red and Mediterranean seas, cholera died out on ship board, before it could be conveyed from Calcutta around the Cape of Good Hope to England. It was formerly conveyed across Central Asia to Russia, because Russia had monopolized almost all the trade with the Asiatic tribes, and we know that cholera follows the lines of commerce. This Russian trade extended more especially from the towns of Astrachan on the Volga, and Orenburg on the Ural river, down to the very borders of Hindoostan. There is hardly a tent or house in all Central Asia, which is not supplied with Russian articles. No less than 3000 camels are employed in carrying iron pots alone, from Orenburg, and one company of traders employ 27,000 camels. Of course cholera has often travelled and will continue to travel overland from India to

Russia, as long as this extensive trade is maintained, and the towns of Orenburg and Astrachan will not only be the most exposed, but will also be the first to contract the disease. This was so well known in 1832, that the Asiatic cholera was called the Russian cholera when it reached the borders of England.

Another great line of cholera-travel towards Europe, is up the Persian Gulf, through Persia to the Caspian Sea, and Astrachan in Russia. These three great routes were illustrated by very carefully prepared maps.*

As cholera always, or at least very frequently, moves along the great lines of travel and commerce, the question whether it is communicable from one person to another, is a most important one, and this question is now considered as having been definitely settled by the experiments of Thiersch, in 1854, and the corroborative ones performed in England during the past season by Burden Sanderson, and Thudichum.

In repeating the experiments of Thiersch, these latter gentlemen discovered that when the cholera matter had killed one series of mice, another series which devoured the dead bodies of the former, were seized with the disease in equal virulence, the mortality ranging as high as 57 per cent.

Carrying on the experiments to a *third series* of mice, a mortality of 50 per cent. ensued.

It is very interesting to note that these experiments succeeded in August and September, but always failed in November, owing doubtless to the low temperature which prevails at that time. There seems to be some relation between this result and another great fact, that cholera almost always dies out in the fall of the year, or rarely continues during the winter. The author then took up the subject of microscopic fungoid growths, as connected with the causation of cholera, giving special prominence to recent observations made by the Germans, which would seem to prove a vegetable origin. It ought to be added,

* The author has very kindly placed at our disposal, many of the more important maps showing the course and distribution of the principal epidemics, which, starting from India and Hindoostan, have travelled through Europe, and from thence been transported to the United States, and spread itself, by means of military conveyance, to various army stations and Indian tribes. We regret that a want of space precludes us from availing ourselves of his generosity. These society Reports follow each other in such rapid succession, and demand so much of our space for mere *printed* matter, that anything but the most meagre illustration in the way of engravings is necessarily precluded, both on account of want of room and time. By frequent reference to ordinary maps, the careful reader will be enabled to follow the author's interesting researches, and thus, though perhaps less satisfactory, supply the want of illustrations.

that the ordinary vibriones have frequently been discovered in large quantities in the rice water evacuations, even while yet contained in the small intestines, showing merely that there is a remarkable proneness to decomposition of the intestinal contents. English observers, as a rule, have not placed much stress upon these microscopic bodies, being content to destroy their pernicious effects by liberal applications of carbolic acid, permanganate of potash, and other disinfectants.

In our own country we have had but little cholera this year, still that little has been in interesting quarters. Last season, viz., 1866, it went from New Orleans, up the Mississippi to the Arkansas, to Little Rock, thence to Fort Smith, from thence to Fort Gibson and Fort Arbuckle. It again broke out in all these places this spring (1867), and also at Helena and Memphis.

At Fort Gibson it commenced in June, and soon rose to the high mortality of 25 per day in that little place, and was also handed over to the Cherokee and Creek Indians, in that neighborhood.

Fort Gibson has an historical interest in connection with cholera, for the disease has prevailed there no less that four different times. It broke out in New Orleans in October 1832, and again in May 1833, and was carried to Fort Gibson in the third quarter of the year 1833, and destroyed 170 persons.

The cholera of 1848 began in New Orleans in December, and was carried up the Arkansas river in steamboats, and reached Fort Smith in the second quarter of 1849, and from thence was again carried to Fort Gibson, where 181 cases occurred in the U. S. army in July and August of 1849. In May 1851, cholera was again carried to Fort Smith by two companies of the 5th infantry, who came from Corpus Christi, Texas, with cholera.

It was also carried last year from St. Louis and Jefferson Barracks near it, up the Missouri river to Fort Leavenworth, where it again broke out this spring, and was carried to Forts Riley and Harker, and from thence to the new town of Ellsworth, which at that time was only six weeks old; but cholera reached it unerringly, because soldiers, rail-road laborers, and others coming from infected districts, passed through there with the disease.

This is a new line of travel for cholera to take from Fort Leavenworth. Formerly it was always carried northwest along the great Oregon trail to Forts Kearney and Laramie, and thence across the Rocky mountains.

Fort Leavenworth has great historical interest in connection with cholera. It prevailed there in 1833, in June, 1849, July, 1850, June and July, 1851, May, 1852, June, 1854, and again in 1866 and 1867. It has regularly been brought there from St. Louis, and was handed along the great line of travel to Oregon, along the Platte River to Fort Kearney, and from thence to Fort Laramie, and from thence to the Pacific coast. The reason that it has so often been brought to Fort Leavenworth is, that this place is not only on the great line of emigrant travel to Oregon, New Mexico, and the great plains, but also used to be the great depot for supplies, and a rendezvous or starting-place for all the United States troops going west. Now the line of travel is somewhat diverted to the Pacific Railroad.

In 1854, over 1000 emigrants died of cholera on the road, before they reached Fort Kearney, and many Indians, who loitered along the emigrant road from curiosity, and for the purpose of begging, paid a terrible penalty.

Thus, there have been no less than eight epidemics of cholera at Fort Leavenworth, all due to its intercourse with St. Louis. In 1866 there were 3500 deaths from cholera in that city, and this year 482. The cholera of 1832 did not reach Fort Leavenworth till 1833, but it reached the Sac and Fox Indians, then in Iowa, from Fort Crawford, near Prairie du Chien, as early as August, 1832, and from Fort Armstrong, on Rock Island, in September, 1832. The Black Hawk war was then going on, and the U. S. troops contracted the disease (then coming down from Canada) at Detroit, carried it to Fort Dearborn, or Chicago, and from thence to Forts Crawford and Armstrong, and gave it to the Sac and Fox Indians.

An outbreak of cholera has just occurred at the Philadelphia Navy Yard, in which there were forty deaths in the receiving-ship, traced to new recruits enlisted with incipient cholera.

An outbreak at Havana is now reported.

An English troop-ship, the Himalaya, direct from Malta, has brought the disease to Quebec. The Himalaya took two soldiers on board with premonitory diarrhœa or incipient cholera. They were the first cases of cholera which occurred on board the Himalaya. The disease died out on the long voyage from Malta to Quebec, but as infected articles possibly had been retained on board, Dr. Marsden carried out the strictest sanitary measures against her.

Professor N. S. Davis, of Chicago, has just published an article, in which he takes the ground that true Asiatic cholera can originate in any city and any country, irrespective of importation. That the same causes which now are acknowledged to give power and activity to the infection, are capable of originating it anywhere. These causes are, high temperature, moisture, accumulation of decomposable animal and vegetable matter, and absence of proper ventilation and drainage. He denies that the epidemic of 1866 in Chicago, either in its beginning, progression, or decline, could be traced to any influence from the importation of persons or goods from other localities.

It is sufficient to say that the causes of common cholera prevail every year, but we never have Asiatic cholera in this country, except after it has occurred in Europe, that it always prevails in the East before it does in the West, and that it is easy to confound common cholera with Asiatic cholera.

The distinction between common or country cholera and the true epidemic pestilence, was made, even in Hindoostan, as early as 1817. In common cholera, which is allied to diarrhœa, cholera-morbus, etc., the loss is six or seven per cent., while in true Asiatic cholera, the loss is sixty to seventy per cent. It is a significant fact, that Dr. Davis has been remarkably successful in the treatment of what he supposes to be true cholera.

No one denies that diarrhœa, cholera-morbus, cholera-infantum, and septic cholera prevail very largely in the dirty portions of all great towns, in the summer. But neither of these diseases is true Hindoostanic or Asiatic cholera. The English cholera often approaches the Asiatic in severity, at least in appearance, but the majority of cases recover, and the more experienced English physicians do not confound these diseases, except, perhaps at epidemic times. But Dr. Davis' article, and another in the *Boston Medical Journal*, are interesting, as proving how closely ordinary cholera sometimes resembles its more malignant prototype.

### *Cholera in Europe in 1867.*

Cholera prevailed at Warsaw, in Poland, between June and August of this year. There have been about 4000 cases and 2000 deaths.

There have been many cases in Switzerland, all traced to direct importation from Italy.

At Rotterdam (Holland), there were 18 or 20 cases a-day in the beginning of September, but by the 21st, it had decreased to two or three per day. It has remained at Rotterdam since 1865, or rather has broken out again in the summers of 1866–67. I do not know how it reached Warsaw, but Drasche, the celebrated historian of cholera, says it was conveyed direct from Rome to Zûrich in Switzerland, by a family which fled from the former place: a child, which had already been sick with premonitory diarrhœa, was the first attacked, then the washerwoman, then a relative, then a friend who often visited the house. There were 591 cases in Zûrich this summer.

The rest of Europe has escaped this year, with the exception of Italy, Tunis, and the islands of Sicily and Malta. Sicily escaped in 1865, in consequence of a strict quarantine, but an insurrection breaking out in 1866, Italian troops were sent from Naples and other parts of Italy, and carried the cholera with them. In fact, this is the third year in succession that Naples has been visited by cholera, and the causes are obvious. There is not a street or lane in which putrid exhalations and pestilential smells are not emitted from many sewers and choked conduits, poisoning the air, and even rendering it necessary to rinse the mouth frequently, to get rid of the disagreeable taste. The water is also bad.

From Naples it was carried over to Palermo in Sicily, by Italian troops. This was so manifest, that the soldiers were charged with having poisoned the wells, fountains, etc., and much popular indignation was excited against them. In Sicily there were no less than 12,000 cases and 7000 death in two weeks, and 60,000 people left the island. In Italy, last summer, the ravages of the disease were very great, no less than 63,000 cases and 32,000 deaths having occurred in a few months. The same scenes of prejudice and superstition were enacted in Italy as in Sicily, aided by the machinations of the reactionary party against Victor Emanuel, especially by the old Bourbon party, and, it is said, by some of the priests whose religious establishments had been broken up.

The people would not only not drink water, neither would they use it for washing purposes. Doctors, soldiers, apothecaries, nurses, and witches, were accused of carrying the cholera about in powders and ointments, scattering it in the air, and putting it in the water. The use of disinfectants probably led to this malicious charge, and the well known malign influences of bad water, especially that contaminated with matters from the sewers, led ignorant people to believe, and designing villains to charge that the drinking water had been poisoned. Many apothecary shops were destroyed to get at the ointments and powders which were supposed to disseminate the disease. Railroad trains and the mails were stopped, and, finally, the dead were left to rot in the houses from whence all the rest had fled. The soldiers were obliged to break open these houses and bury the offensive corpses. Finally, the dying refused to reccived the sacred wafer from the priests, fearing that it might be poisoned, and rejected both food and medicine from soldiers and priests. Soldiers, physicians, apothecaries, and supposed witches were mobbed as cholera-agents. The noble and zealous often showed as much ignorance as the superstitious. Thus, when cholera broke out in Albano, Cardinal Altieri, the bishop of that place, started at once from Rome, took all the money he could raise, stripping his palace of all clothing, bedding, and food, and took two physicians with him. When he reached Albano, he addressed the people at once, carried the sacred host barefooted through the streets, administered the sacrament to all, gave away all his linen and beds, took no sleep, and ate coarse food. He, of course, died in three or four days. So strong was the belief that the soldiers and the authorities caused the cholera, that a brigand, named Palma, ordered the professors and prefect of the town of Rossano to cause the disease to cease instantly, or he would come down with 4000 men, and burn and destroy everything before him.

A Society of the Sacred Heart of Jesus was formed to prevent cholera; each member to wear a cross cut out of red woollen, and surmounted by a little cross, the whole to be fastened upon a square of white woollen, with the inscription, "Stand off, the heart of Jesus is with me." Bishop Fetici, of Parma, granted forty days' indulgence to all who would wear the badge and repeat certain prayers daily.

How different has been the conduct and success of the English authorities, especially in and near Bristol, the home of Dr. Budd. They attended to the things which were before their eyes, and under their noses, and seemed to care little for the air above or the waters under the earth, except that used for drinking. Thus Pill, a little town five miles from Bristol, England, with 1800 regular inhabitants, became fearfully overcrowded by sailors and railroad laborers. There had been twenty-five cases of cholera. The town was very filthy, and had a ditch and brook filled with refuse matter from sewers; many privies emptied into it by open drains. Many houses had no privies, but their inhabitants used the banks above the brook for this purpose, so that it was absolutely covered with fœcal matter. All the cases could be, and were traced to direct contagion. There was no trifling with a few teaspoonfuls of carbolic acid or a handful or two of chloride of lime, but all the privies and dunghills were thoroughly disinfected, one-half ton of sulphate of iron was put in the ditch, and the surface covered with chloride of lime. Where the brook emptied into the village, one hundred pounds of sulphate of iron was thrown in every morning; a strong solution of sulphate of iron was put in every drain, and carbolic acid, one-half pint to a bucketful of water, followed after. The privies were washed down with a solution of carbolic acid, fumigated with chlorine, and covered with chloride of lime and charcoal powder. Filthy greund was watered with solutions of carbolic acid, and covered with Calvert's powder. Infected houses were fumigated, whitewashed, and the floors scrubbed with a solution of permanganate of potash. All bedding and linen, soiled with cholera discharges, were, as far as possible, destroyed. Nurses were sent down from Bristol, and went to every house where cholera existed, and taught the inmates how to use disinfectants in bed-pans, close stools, chamber pots, etc.

Cholera discharges had been thrown upon a heap of refuse near the principal well of the town, this was so drenched with carbolic acid, that an accidental rain rendered the water undrinkable. Depots were established where medicines, beef-tea, milk, and ice could be obtained, and schoolmasters and clergymen were supplied with cholera medicine. It required four days to carry out these arrangements. During the first two days, fourteen fresh cases occurred; upon the fourth day, only seven new cases were noticed, and many of these had been previously infected. The last case occurred on the twelfth day after these proceedings were established. In twelve days, the epidemic was at an end.

As Malta is in the direct line of travel from Mecca to Alexandria, and many Mohammedans live on the island and often go on pilgrimages to Mecca, the disease now prevails there very frequently. In the week before October 4th, 1867, one hundred and forty cases, and ninety deaths had occurred.

### *Cholera in India in 1867.*

In Hindoostan, the home of cholera, what is now graphically called the "Pilgrim nuisance" has been forced anew upon the medical and other authorities. The *London Lancet* of July, 1867, says: "There never was, perhaps, a more forcible illustration of the doctrine that cholera travels along the lines of human intercourse, than that supplied by recent occurrences in April, May, and June, 1867, at Hurdwar and its vicinity. One of those well-known great native gatherings took place there this year, in April, 1867. Cholera appeared; the vast assemblage broke up, and dispersed towards their homes and spread the disease along the whole line of their route. From Hurdwar, as a centre, did the disease radiate outwards in the diverging lines taken by these natives."

The Indian correspondent of the *British Medical Journal*, of June 8th, 1867, writes: "The returning Hurdwar pilgrims seem to be carrying the cholera poison with them in all directions. Despite every precaution taken to keep them out of the English military station at Umballa, which is the first post north-west of Hurdwar, and scarcely twenty miles away, and that by means of a cordon of police and the troopers of the 11th Bengal cavalry, two infected pilgrims managed to get into the Bazaar of the 94th regiment of white troops. In consequence, some thirty cases of cholera soon occurred, including three medical officers, one lieutenant, some white soldiers, and many native troops and camp followers. Up to April 19th, 1867, it is known that 269 cases of cholera occurred in one column of pilgrims in the short stretch between Hurdwar and Umballa, and many more deaths happened in this and other columns which pursued this and other directions."

The great towns of Lodiana and Lahore which, like Umballa, are on the grand trunk road to the north-west, were reached next in order. From Lahore, cholera spread north-west in a direct line along the grand trunk road to Attock and Peshawur. In the London *Medical Times and Gazette*, we read: "During the present epidemic (1867), Peshawur, the advanced post of north-western India, has suffered most severely, though cholera is now widely disseminated throughout India, and exists at most of the military stations in Bengal. Ten per cent of the troops at Peshawur have already been attacked, and fifty-eight per cent of these have died."

At the present time, the English Government maintains a regular standing force between Hurdwar and Peshawur, of more than 12,000 men. A large English garrison is kept at Amballa or Umballa, the nearest point to Hurdwar, and at Lodiana, a little farther north. From Peshawur, the cholera of 1867 will almost certainly be taken to Cabul, thence to Balk, and finally to Bokhara, which latter place our friends the Russians are now beseiging, or have already taken. The cholera may reach the Russians there, and they may carry it back to Khiva, and thence to Astrachan and Orenburg, where it has always beeu previously conveyed by the Asiatic and Tartar tribes. The above facts are particularly interesting, because cholera has always been conveyed to Central and Western Asia, to Persia, and to Russia, by this same route through Attock and Peshawur. In fact, if it is conveyed by persons, and not solely by winds, it can go in no other direction.

The province of the Punjaub, the extreme north-western province of Hindoostan, which has Hurdwar on its south-eastern boundary, and Peshawur, the border town, or extreme advanced post of all Hindoostan, on its north-western line, is enclosed on all its northern and eastern sides, by the Himalaya mountains, extending in one unbroken line for nearly 1000 miles in length, and about 80 in breadth, forming a continuous pile of precipices, rocks, snow, and ice.

Nothing resembling a wagon, or even ordinary beasts of burden, such as horses or oxen, can pass this barrier. Goods can only be carried on the backs of sheep and goats, and thus a scanty trade is carried on between Hindoostan and Thibet. The first and only break or pass in the Himalaya mountains is at the town of Attock, where the Cabul river makes its junction with the Indus.

The Province of Punjaub and the town of Attock, have great historical, commercial, and medical interest. Almost all the invasions from the time of Alexander the Great—336 B. C., have taken place along the line of the Cabul River, through the town of Attock. Almost all the trade of Hindoostan, with Persia, Independent Tartary, Central Asia, and Russia, almost every epidemic of cholera has followed the same route of travel and conquest, and one of the great nurseries of cholera exists at Hurdwar, just below the Southern boundary of the Punjaub.

I am not quite certain that the first great modern epidemic of cholera, that of 1817, escaped by this route. It probably did, but up to the present time no European has ever been able to go from Cabul to Balk, Bokhara, and Khiva, except in disguise, and the sources of information are necessarily scanty.

But in 1827, it destroyed 30,000 persons in Lahore, and passed from there to Cabul, over the Himalaya mountains, and from this last city, which is a great emporium for the merchandize of Russia, it was tranferred by caravans through Balk, Bokhara, and Khiva, to the Caspian Sea and Russia. At Cabul the two great lines of travel from Persia and Russia meet, and at Cabul the great line of cholera coming up from India divides, the one line leading to Persia, through the towns of Herat and Teheran, and finally to Astrachan; the other leads through Balk, Bokhara, and Khiva, to Orenburg, in Russia.

Another writer says: "Hurdwar is a great mart of commerce, and a celebrated place of Hindoo pilgrimage. In the month of April the pilgrims perform their ablutions in the sacred Ganges; and great numbers of merchants follow, forming one of the largest fairs known in Hindoostan. In 1807, no less than two millions of strangers attended them. In 1783, we are told by many authorities, that about one and a-half million dervishes assembled at Hurdwar, to celebrate a religious festival of peculiar popularity, and that in eight days 20,000 of them died of the cholera." It is perfectly evident from the above, that if cholera originates anywhere in India and reaches the neighborhood of Hurdwar, it will be strengthened there every year in April, and that every *twelfth year* this influence will be quadrupled, or more. Every year that cholera originates at Hurdwar, or is brought there, it will be conveyed northwestward through Attock and Peshawur to Cabul, and then will proceed due westward to Persia, northward to Balk, Bokhara, Khiva, to the nearest Prussian commercial towns, which are Astrachan and Orenburg.

Roberts says: "The largest of all the Indian fairs is held at Hurdwar, at the time of the vernal equinox. From 200,000 to 300,000 persons congregate there every year, and *every twelfth year* the number of pilgrims, merchants, and visitors frequently exceeds one and a-half millions. This fair is a focus for the produce of all India, from Calcutta in the south-east, to Bombay in the south-west, and all the intermediate provinces: also of Cabul, Afghanistan, Persia, Arabia, and Independent Tartary. Horses, cattle, camels, jewelry, Persian dried fruit, spices, shawls, cotton goods, cutlery, etc., are the principal articles of trade."

Of all the great seaport towns of Hindoostan, as connected with the transportation of cholera, Bombay has excited most interest since 1865; far more, in fact, than Calcutta used to. It seems well proven, that our last great epidemic, viz., that of 1865, was conveyed from Bombay to Makulla, on the southern Arabian coast, thence up the Red Sea to Mecca, thence to Suez, Cairo, and Alexandria, to the Mediterranean; and from Alexandria to Constantinople, Malta, Marseilles, and Southampton, England.

Bombay should have excited much more attention long ago than it has yet received.

The cholera of 1817 started from Allahabad, at the junction of the Ganges and Jumna Rivers, reached the Marquis of Hasting's army, just below in the Bundlecund, was carried down to Nagpoor, and from thence to Poonah and Bombay, which it reached early in 1818, by means of troops passing to and from Bombay and Nagpoor to coöperate with the Marquis of Hastings.

There were nearly 16,000 cases of cholera in Bombay, from August 1818, to February 1819. In 1821, the Presidency of Bombay was again the seat of the disease, in its most mortal and malignant form; and it was carried over to the Persian Gulf by a convoy of English troops.

It returned again to Bombay in 1825, in great severity, and caused great alarm. Large cholera hospitals were built, and immense quantities of wood, tar, and gunpowder were burned, and large quantities of vinegar used as disinfectants. The epidemic of 1821 progressed up the Persian Gulf to Bushire, thence crossed Persia by way of Shiraz, Ispahan, and Teheran, to Tabrez and Tiflis, and from thence to Astrachan, in Russia, which it reached in 1823. At Astrachan the precautions of the Russian Government were so efficient that the disease was stayed, and did not reach that place again until the 19th of July, 1830, seven years afterward, when the old belief in contagion and infection having died out, the disease was allowed to spread to Russia, and also to Europe.

The cholera of 1829 at Bombay, was conveyed over the same route, but did not stop at Astrachan, for it was carried through to Poland and Prussia, to Hamburg, and from thence to London.

During the last year renewed attention has been paid to the frequency and extent of Hindoo pilgrimages, and their influence upon cholera. The river Ganges has been found to be dotted with holy places, from its mouth in the Bay of Bengal, to its source in the Himalaya mountains, at Hurdwar. At the mouth of the Hoogly, below Calcutta, is situated Saugar Island, the extreme point of which is considered by the Hindoos to make the junction of the Ganges with the sea, and is accordingly esteemed as one of the holiest spots in India. At a certain season of the year they flock thither in great numbers, for the purpose of bathing and offering sacrifices. There is much reason for the belief that the first great cholera originated here, and was carried up the Ganges, both to Calcutta and Jessore, although both of these places are filthy enough to generate cholera within their own limits. Several great festivals take place annually at Calcutta.

About 150 miles north of Calcutta is Gaya, the birth-place of Budda, and the scene of Vishnu's incarnation; a place annually visited by vast number of pilgrims. Near Patna is the remarkable mountain Junghera, rising like an island from the Ganges. It was formerly considered the holiest spot along the whole river, so that thousands of boats and larger vessels were constantly to be seen there, as many Hindoos thought they could not die in peace without visiting it.

Benares, still further North, is the holy city of the Hindoos, of far greater sanctity to him than Mecca to the Musselman, or Jerusalem to the Jews, for here Mahadeo, the god of the Creative Principle made his last appearance on earth. It is so particularly sanctified, that all who live within a circuit of five miles, or visit it, go to Heaven, whether they wish to or not. The daily number of devotees on the banks of the river is 50,000, and at least 300,000 or 400,000 arrive annually at the great festival of Mala, in honor of Mahadeo; and the river is black for miles with the bathers' heads. The streets are both dirty and ugly. Many of them are so narrow that there is scarcely room for a palanquin to pass.

The Ganges waters near Benares is so holy that it is carried great distances. Bayard Taylor found the road swarming with pilgrims, each carrying his two jars of Ganges water to his home. In one afternoon he passed thousands and thousands of the lowest and poorest castes thus employed.

Allahabad is 76 miles North of Benares, near the junction of the Jumna and Ganges. It is called the city of Allah, or the city of God, because it is believed that a third invisible river flows direct from Paradise and joins the Ganges here. The festival at Allahabad takes place in February. Bayard Taylor found the road thronged with pilgrims returning from it, and most of the women, as well as men, carried large jars of Ganges water suspended from poles. We can now for the first time understand the almost inconceivable rapidity with which cholera spreads along the Ganges, both up and down, when an epidemic once commences. It must also be remembered that *the influence of these pilgrimages is increased from four to ten-fold every twelfth year.* But these religious festivals occur not only along the Ganges, but also throughout all India, and not only every year, but almost every month in the year. Thus every temple, like those at Juggernaut and Conjeveram, has a festival on the anniversary of its dedication, which lasts ten days, and people assemble from all parts of India.

Great attention has been paid by the English authorities to the sanitary condition of the pilgrims to the great temples of Juggernaut and Congeveram. Cholera used to occur at both these places, and notably so on the twelfth year. Both these places have great historical interests in connection with cholera. The great epidemic of 1781, recorded by Curtis, the only one which has been accurately described previous to 1817, originated in the neighborhood of Juggernaut, at the annual festival which takes place early in March, when the moon is of a certain age. Colonel Pearse's force of 5000 men was assaulted at Ganjan, only a few miles below Juggernaut, on March 22, 1781, in the same terrible manner as were the forces of the Marquis of Hastings in the Bundlecund, *three times twelve*, or thirty-six years subsequently, viz., in November, 1817. The onset commenced with almost inconceivable fury. Men previously in perfect health dropped down by dozens, and those less severely attacked, were generally past recovery in less than twenty-four hours. The cramps of the limbs and body were dreadful, and the distressing vomiting and purging were present in all cases. Besides those who died, over 500 sick accumulated in the hospital in the course of a few days, and in a short time more than one-half of the army was on the sick list. This epidemic forced its way up some 250 miles to the North-east to Calcutta, and occasioned a great mortality among the native inhabitants, and then pursued its path still further Northward, but doubtless owing to the scantiness of the European population at the time, all attempts to trace its further progress are said to have proved fruitless; still it is well to notice that the third period of *twelve* years, or thirty-six years after, will bring us to the great epidemic of 1817; and the seventh period of twelve years, or eighty-four years, subsequently falls upon the last epidemic of 1865, a fact which I believe I was the first to notice.

The thanks of the Society were tendered to Dr. Peters for his able and interesting resume.

Dr. M. Herzog remarked that Thiersch had recently established the interesting fact, that cholera was not communicable from one person to another in less than twenty days.

The experiment of Pettenkofer proved that sporules of cholera could not exist where there were a plentiful supply of pure water. In houses supplied with water-closets, no cases of cholera occurred in New York city in 1867. There were two exceptions to this, but it was found that in these cases the water-closets were defective.

He considered that the cause of the periodical increase of cholera had a deeper cause than the twelfth year theory, and was to be explained by the prevalence of rain and subsequent dryness forming a subsoil stratum of moisture. He considered that, during 1867, we were protected from the spread of the cholera by the plentiful and continuous rains which occurred during the whole summer, but as this will tend to establish a great rise in the subsoil water, he sincerely believed that we will be more in danger of cholera in 1868 than we have been in 1867.

Dr. Noyes, of the Committee on Portraits, reported favorable progress. A photograph of the Staff of the New York Hospital had been secured, and Dr. S. Katz had presented a valuable collection of portraits of the celebrated physicians and surgeons of France.

The Society then adjourned. M.

